# Development of a safer laboratory vervet monkey model for the study of human African trypanosomiasis

**DOI:** 10.4102/ajlm.v3i1.100

**Published:** 2014-10-29

**Authors:** Maxwell Waema, Naomi Maina, Simon Karanja, Beatrice Gachie, Maina Ngotho, John Kagira

**Affiliations:** 1 Institute of Tropical Medicine and Infectious Diseases, Jomo Kenyatta University of Agriculture and Technology, Kenya; 2 Biochemistry Department, Jomo Kenyatta University of Agriculture and Technology, Kenya; 3 Animal Science Department, Institute of Primate Research, Kenya; 4 Department of Land Resources Planning Management, Jomo Kenyatta University of Agriculture and Technology, Kenya

## Abstract

**Background:**

There are three subspecies of *Trypanosoma brucei*: *T. b. gambiense, T. b. rhodesiense* and *T. b. brucei*. The first two are infectious to humans, whilst *T. b. brucei* is not. Identifying an animal model of *T. b. brucei* that mimics human African trypanosomiasis (HAT) would enable researchers to study HAT without subjecting themselves to undue risks such as accidental infection.

**Objectives:**

This study assessed the sequential clinical, parasitological and haematological changes in vervet monkeys infected with *T. b. brucei*.

**Methods:**

Three vervet monkeys were infected with a 10^4^ inoculum of *T. b. brucei* (isolate GUTat 1). Late-stage disease was induced by subcurative treatment with diminazene aceturate 28 days post-infection. The animals were treated curatively with melarsoprol upon relapse. Parasitaemia and clinical signs were monitored daily and, at weekly intervals, the monkeys’ blood and cerebrospinal fluid (CSF) were sampled for haematology and parasitosis assessments, respectively.

**Results:**

The first-peak parasitaemia was observed between seven and nine days post-infection. Clinical signs associated with the disease included fever, dullness, pallor of mucous membranes, lymphadenopathy, splenomegaly and oedema. Late-stage signs included stiffness of joints and lethargy. The monkeys developed a disease associated with microcytic hypochromic anaemia. There was an initial decline, followed by an increase, in total white blood cell counts from early- to late-stage disease. Trypanosomes were detected in the CSF and there was a significant increase in white cell counts in the CSF during late-stage disease. Infected vervet monkeys displayed classical clinical symptoms, parasitological and haematological trends that were similar to monkeys infected with *T.b. rhodesiense*.

**Conclusion:**

The *T. b. brucei* vervet monkey model can be used for studying HAT without putting laboratory technicians and researchers at high risk of accidental infection.

## Introduction

*Trypanosoma brucei* (*T. brucei*) is a protozoan parasite, of which there are three subspecies: *T. b. gambiense, T. b. rhodesiense* and *T. b. brucei*. The first two subspecies are infectious to humans, causing human African trypanosomiasis (HAT) (also known as sleeping sickness),^1^ whilst *T. b. brucei* only causes disease in animals. The disease has two recognised stages: the early (haemolymphatic) stage when parasites appear in the blood and the late (encephalitic) stage when the central nervous system is involved.

*T. b. rhodesiense* is found in eastern and southern Africa and causes an acute infection. The first clinical symptoms are observed a few weeks or months after initial infection – a result of the parasite invading the central nervous system (CNS). *T. b. gambiense* is found in west and central Africa and causes a chronic infection. The parasite can be present in the body for months or even years without causing severe symptoms. For the majority of patients infected with *T. b. gambiense*, the disease is already in an advanced stage by the time symptoms emerge, with parasites having affected the CNS.^1^

HAT remains a neglected disease of major health and socioeconomic consequence in sub-Saharan Africa,^1^ with an estimated 60 million people in 36 countries at risk.^2^ The management of the disease remains a major challenge due to poor diagnostics and treatment regimens, most of which have been used for decades.

Research and development of diagnostics and new drugs normally rely upon the use of laboratory animal models, principally mice and monkeys, which mimic human disease. Six accidental, laboratory-acquired cases of *T. b. rhodesiense* have been reported.^3,4,5^ Rather than identifying an animal model of a dangerous human pathogen, a safer option would be to identify a non-human pathogen that mimics HAT. Unlike *T. b. rhodesiense, T. b. brucei* is unable to infect humans, but mimics the pathogenesis of human-infective *T. b. rhodesiense* in infected animals.^6^ Thus, an animal model of *T. b. brucei* that mimics HAT would enable researchers to study the disease without subjecting themselves to undue risks. Given that the vervet monkey disease model of *T. b. rhodesiense* bears a close relationship to HAT,^7^ the authors hypothesised that infection with *T. b. brucei* could provide similar disease progression. These monkeys may provide an excellent opportunity to investigate controlled laboratory studies on serum and cerebrospinal fluid (CSF) samples, which would also allow for identification of potential biomarkers of the disease stages.

This study was designed to determine the clinical, parasitological and haematological profile of vervet monkeys infected with *T. b. brucei* and to determine whether a *T. b. brucei* model would be a suitable and safer alternative to the vervet monkey model of *T. b. rhodesiense*.

## Research methods and design

### Trypanosomes

The *T. b. brucei* GUTat 1 isolate was used. The isolate was obtained from the International Livestock Research Institute Biobank, passaged three times in mice and preserved at the Institute of Primate Research’s trypanosomes cryobank. The cryopreserved isolate was thawed and injected into donor Swiss white mice for expansion. At peak parasitaemia, the mice were euthanased with CO_2_ and their blood was harvested by cardiac puncture. The blood was serially diluted in phosphate saline glucose to a final concentration of 10^4^ trypanosomes/mL.

### Drugs

The trypanocidal drugs used in this study were diminazene aceturate (PHARMA Links, India) and melarsoprol (Arsobal^®^, Specia, France).

Diminazene aceturate is an aromatic diamidine used as treatment for livestock trypanosomiasis and is also effective against early-stage *T. b. gambiense* and *T. b. rhodesiense* infection.^7^ In monkey models of HAT, the drug clears bloodstream parasites but is unable to clear parasites in the CNS since it does not cross the blood-brain barrier. Its mode of action involves interference with RNA editing and trans-splicing and it inhibits AdoMet decarboxylase in trypanosomes, resulting in the reduction of spermidine content and the elevation of putrescine levels in the parasite.

Melarsoprol is a trivalent arsenical compound used for the treatment of late-stage sleeping sickness. The mode of action involves inhibition of trypanothione reductase in trypanosomes. Melarsoprol can cross the blood-brain barrier and can thus clear trypanosomes that have crossed into the brain parenchyma. In HAT monkey models, melarsoprol is used for curative treatment in late-stage disease.^8^

### Experimental animals

Five wild-caught vervet monkeys, each weighing 2–4 kg, were obtained from the animal unit colony of the Institute of Primate Research. Prior to experimentation, the monkeys underwent quarantine for 90 days, during which time they were screened for zoonotic diseases as well as ecto- and endoparasites. Because of the evidence of minor strongyle infections and mange infestations, they were treated with subcutaneous injections of Ivermectin at dosages of 300 μg/kg for three days. The monkeys were housed in single 90 x 60 x 60 cm stainless-steel cages in a room maintained at temperatures in the range of 23–25 °C. They were fed twice daily with monkey cubes (Goldstar Feeds^®^ Ltd., Nairobi, Kenya), vegetables, green maize and bananas; water was provided *ad libitum*.

### Experimental design

Three monkeys were infected intravenously with 1 mL of the suspension containing 10^4^ trypanosomes of *T. b. brucei* (GUTat 1), whilst the remaining two monkeys were kept as non-infected controls. The infected monkeys were monitored daily for parasitaemia as described in previous studies.^7^ At 28 days post-infection (dpi) the monkeys were treated subcuratively for three consecutive days using intramuscular diminazene aceturate at a dose rate of 5 mg/kg body weight. The monkeys were then monitored for parasitaemia and, on relapse (114 dpi), were treated curatively for four consecutive days with intravenous melarsoprol at a dose rate of 3.6 mg/kg body weight. After 180 days of monitoring, the monkeys were euthanased using Euthatal (20% sodium pentobarbitone).

### Clinical examination and sample collection

The clinical status of the monkeys was monitored daily. At weekly intervals, the monkeys were anaesthetised with intramuscular injections of ketamine hydrochloride (Ketamine^®^, Rotexmedica, Trittau, Germany) at dosages of 10 mg/kg body weight and full physical examinations were conducted. Furthermore, 2 mL of blood were collected weekly by venepuncture of the femoral vein and placed in tubes containing Ethylenediaminetetraacetic acid (EDTA). CSF was collected weekly by lumbar puncture.

### Laboratory analysis

Immediately after blood collection, the haematological assays were performed using an AC3diff T Coulter counter (Miami, Florida, USA). Parasitaemia was scored using the method described by Herbert and Lumsden,^8^ which included the daily use of wet smear detection of microscopic parasites using the rapid matching method. To determine pathological effects, baseline biochemical values were compared to those post-infection. Normal ranges are not generally used because of the variation found amongst vervet monkeys.^10^

### Disease staging criteria

The stage of trypanosome infection was determined in accordance with World Health Organization criteria and as previously described.^9,11^ CSF was collected by lumbar puncture and examined for the presence of trypanosomes and the number of white blood cells (WBCs). The WBCs were estimated by counting in a Neubauer cell chamber. When cells were < 5 cells/µL, infection was classified as first stage. Late-stage infection was diagnosed when trypanosomes were detected in the CSF and the WBC count of the CSF was > 5 cells/µL.^12^

### Statistical analysis

Data were managed using Microsoft Excel (Microsoft US, version 2007). Descriptive statistics and summary tables were employed for the initial description of the data and the results were displayed in Excel charts. Differences between the means were compared using the Student’s *t*-test and were deemed to have statistical significance at *p* < 0.05.

### Ethical consideration

Prior to commencement of the study, all protocols and procedures used were reviewed and approved by the Institute of Primate Research Institutional Animal Care and Use Committee (IRC/19/10).

## Results

### Clinical signs

All the infected animals developed acute symptoms characteristic of Rhodesian HAT, including fever with a mean temperature of 40 °C, dullness, increased pulse and respiratory rates, pallor of the mucous membranes, enlarged superficial lymph nodes and spleen, raised hair coat, peri-orbital oedema and stiffness of joints. Upon subcurative treatment with diminazene aceturate, most of the clinical signs of disease disappeared within 14 days. However, starting at 42 dpi, one monkey exhibited a general body weakness, sleepiness, ataxia and an arched back when sitting on the cage floor and was euthanased for humane reasons. The other two infected monkeys had relapses of parasitaemia from 114–119 dpi with clinical signs that included stiffness of joints and hind leg paralyses. These signs disappeared within 14 days after curative treatment with melarsoprol.

### Parasitaemia and cerebrospinal fluid parasitosis

**FIGURE 1:**
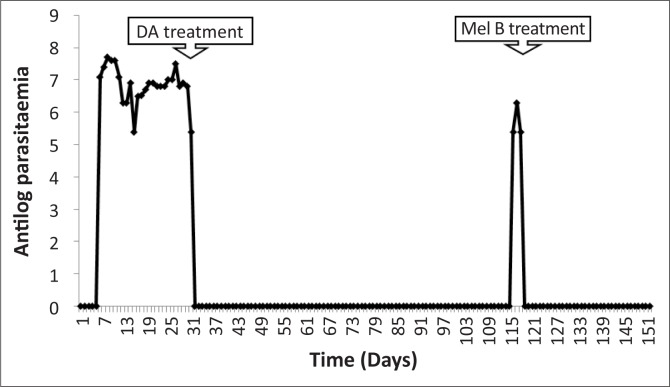
Mean daily parasitaemia of vervet monkeys infected with *T. b. brucei* GUTat 1, indicating the point of subcurative treatment with diminazene aceturate (DA) and curative treatment with melarsoprol (Mel B).

**FIGURE 2:**
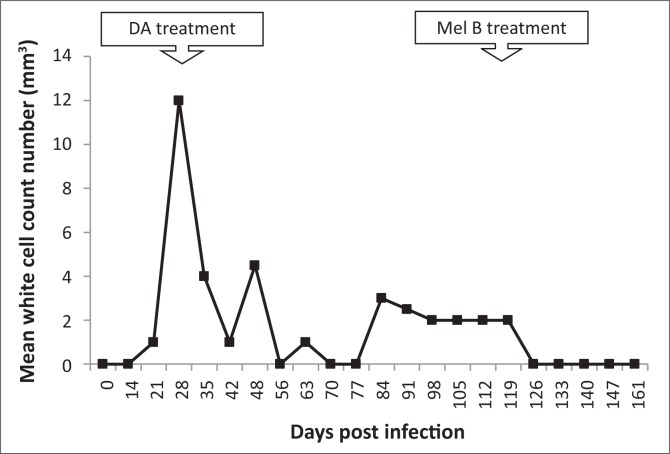
White blood cell (WBC) count numbers in the cerebrospinal fluid of *T. b. brucei*-infected vervet monkeys, showing the point of subcurative treatment with diminazene aceturate (DA) (28 dpi) and curative treatment with melarsoprol (Mel B) (119 dpi).

All experimentally-infected animals had a prepatent period ranging from two to four days. The first parasitaemia peak of 10^7^ trypanosomes per mL of blood occurred between 7–9 dpi (Figure 1). Treatment with diminazene aceturate resulted in clearance of the trypanosomes in the blood. In all three infected monkeys, the parasites relapsed, starting at 114 dpi. Trypanosomes were detected in the CSF of two monkeys on days 28 and 105 post-infection. Treatment with melarsoprol at 119 dpi led to clearance of both the parasitaemia and CSF parasitosis by 123 dpi. There was an increase in CSF WBC counts during the late stage (Figure 2).

### Haematology profiles

#### Erythrocyte changes

**FIGURE 3:**
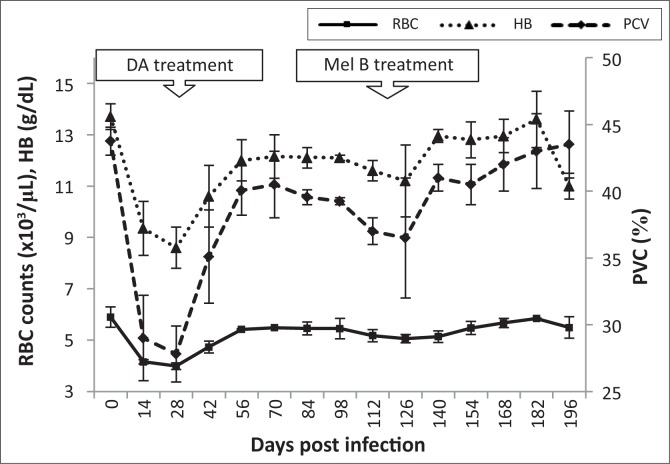
Mean changes in red blood cell (RBC) counts, packed cell volume (PCV) and haemoglobin (HB) of vervet monkeys infected with *T. b. brucei* GUTat 1, showing the point of subcurative treatment with diminazene aceturate (DA) (28 dpi) and curative treatment with melarsoprol (Mel B) (119 dpi).

In the control animals, the mean total red blood cell (RBC) count was 5.4 x 10^6^ cells/μL, packed cell volume (PCV) was 42% and haemoglobin (Hb) was 12 g/dL. Blood count parameters did not change significantly throughout the experimental period. However, by 28 dpi, the infected vervet monkeys had a decrease (*p* < 0.05) in the mean values for the RBC count, PCV and Hb values to 4.7 x 10^6^ ± 0.72 cells/μL, 27 ± 2.05% and 8.2 ± 0.8 g/dL, respectively (Figure 3). After subcurative treatment, the levels increased gradually to those of pre-infection within 14 days post-diminazene aceturate treatment. Blood count parameters decreased again at 98 dpi but recovered and attained the pre-infection levels within seven days of curative treatment with melarsoprol.

**FIGURE 4:**
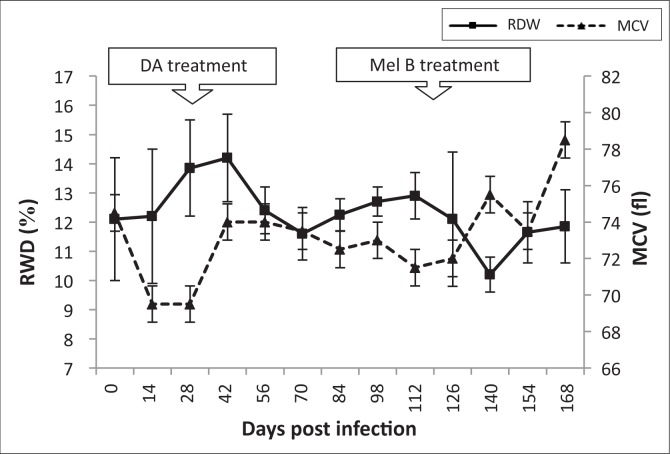
Mean changes in red cell distribution width (RDW) and mean cell volume (MCV) of vervet monkeys infected with *T. b. brucei* GUTat 1, showing the point of subcurative treatment with diminazene aceturate (DA) (28 dpi) and curative treatment with melarsoprol (Mel B) (119 dpi).

**FIGURE 5:**
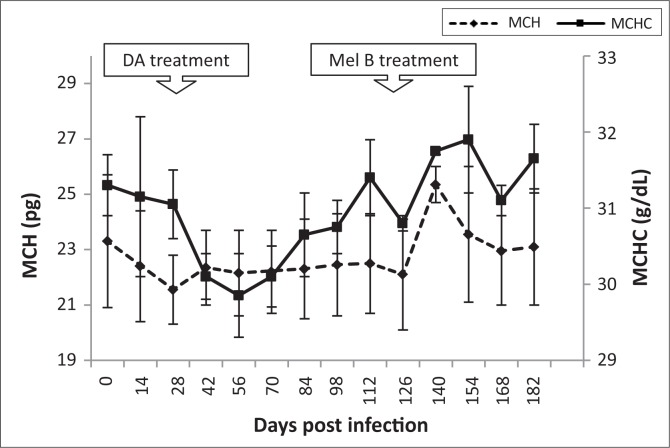
Mean changes in mean corpuscular haemoglobin (MCH) and mean corpuscular haemoglobin concentration (MCHC) in vervet monkeys infected with *T. b. brucei* GUTat 1, showing the point of subcurative treatment with diminazene aceturate (DA) (28 dpi) and curative treatment with melarsoprol (Mel B) (119 dpi).

Red cell distribution width (RDW) increased during early-stage infection and peaked (14.2 ± 1.5%) at 42 dpi. Thereafter, RDW decreased during late-stage disease to normal levels (10.5 ± 0.6%) at 140 dpi. A decrease in mean corpuscular volume (MCV) was observed from the onset of infection until 7 dpi, after subcurative treatment with diminazene aceturate, thereafter returning to pre-infection levels (Figure 4). Mean corpuscular haemoglobin (MCH) decreased 14 dpi from 23.5 to 22.5 pg, levelling off by 28 dpi. Levels appeared stable after melarsoprol treatment (119 dpi), returning to pre-infection levels by 140 dpi (Figure 5).

#### Platelets

**FIGURE 6:**
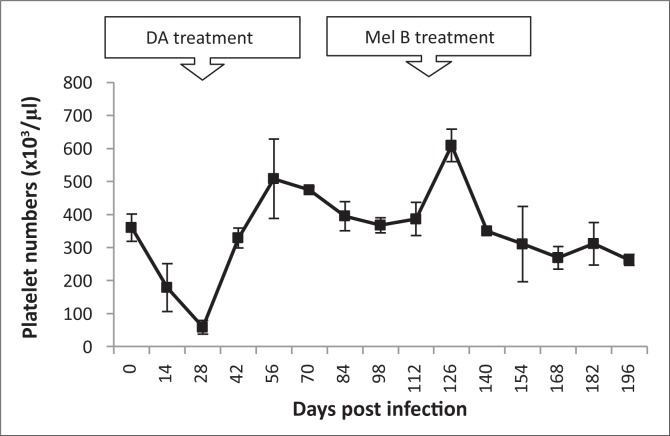
Changes in mean platelet numbers in vervet monkeys infected with *T. b. brucei* GUTat 1, showing the point of subcurative treatment with diminazene aceturate (DA) (28 dpi) and curative treatment with melarsoprol (Mel B) (119 dpi).

Platelet numbers declined after infection, reaching their lowest levels (58.5 × 10³ ± 0.15 cells/µL) at 21 dpi. Levels increased after subcurative treatment with diminazene aceturate, returning to those of pre-infection by 56 dpi (Figure 6). Platelet counts decreased steadily for three weeks before trypanosome relapse in the blood (84 dpi), but stabilised to pre-infection levels (550 × 10³ cells/µL) after melarsoprol treatment (119 dpi).

#### Leucocytes

In uninfected animals, the mean total WBC count ranged from 4.3 x 10^3^ cells/μL to 5.7 x 10^3^ cells/μL and did not change significantly throughout the experimental period (*p* < 0.05). . Mean neutrophil counts ranged from 1.72 x 10^3^ cells/µL to 2.12 x10^3^ cells/µL, lymphocyte counts from 3.6 x10^3^ cells/µL to 4.48 x 10^3^ cells/uL and monocyte counts from 0.3 x 10^3^ cells/µL to 0.26 x 10^3^ cells/µL. None of these counts changed significantly throughout the experimental period (*p* < 0.05).

**FIGURE 7:**
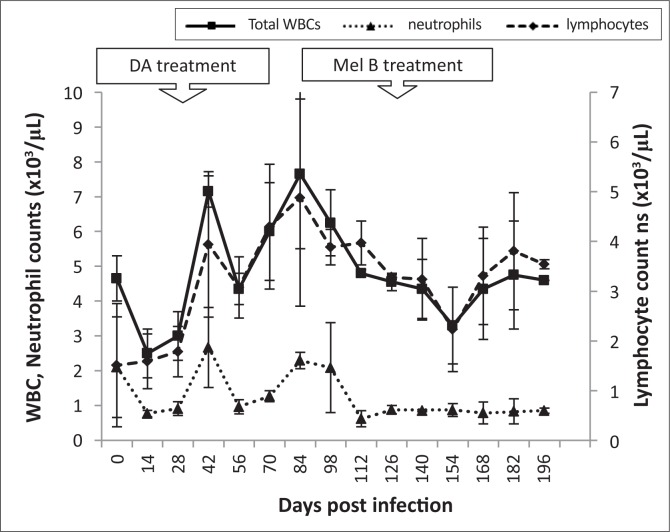
Mean changes in total white blood cells (WBC), lymphocyte and neutrophil counts of *T. b. brucei* GUTat 1-infected vervet monkeys showing the point of subcurative treatment with diminazene aceturate (DA) (28 dpi) and curative treatment with melarsoprol (Mel B) (119 dpi).

**FIGURE 8:**
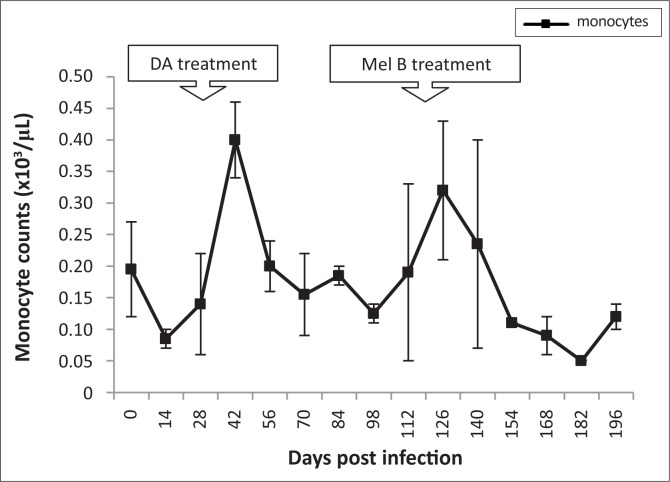
Mean changes in monocyte counts of *T. b. brucei* GUTat 1-infected vervet monkeys, showing the point of subcurative treatment with diminazene aceturate (DA) (28 dpi) and curative treatment with melarsoprol (Mel B) (119 dpi).

In infected animals, total WBC counts declined during early stage infection (4.7 x 10^3^ cells/µL to 2.5 x 10^3^ cells/µL). After subcurative treatment with diminazene aceturate, WBC counts increased and were significantly higher at 84 dpi (7.7 x 10^3^ ± 1.75 cell/µL, p < 0.05) than pre-infection levels (4.3 x 10^3^ ± 1.18 cells/µL). Thereafter, WBC counts declined to 3 x 10^3^ cells/µL by 154 dpi. Both lymphocyte and neutrophil counts followed a similar pattern (Figure 7). Changes in eosinophil and basophil counts were not significant during the course of the disease. Monocyte counts dropped significantly from 0.2 x 10^3^ cells/µL to 0.07 x 10^3^ cells/µL between 0 and 14 dpi. They then peaked at 42 dpi (0.38 x 10^3^ ± 0.04 cells/µL), after which there was a decline. A further increase in monocyte counts was noted, starting at 112 dpi with a peak at 126 dpi (0.3 x 10^3^ ± 0.05 cells/µL) (Figure 8).

## Trustworthiness

To judge from the outcome of *T. b. brucei* infection in vervet monkeys, the observations obtained here appeared to compare well with those arising from *T. b. rhodesiense* and other related studies.

### Reliability and validity of the research

The experimental design of this study is reliable and valid. The procedures used in this research have been tested in other studies as cited in this article.

## Discussion

This is the first study reporting the infection of vervet monkeys with *T. b. brucei*. Human beings and some non-human primates have a trypanolytic factor, which prevents them from being infected with *T. b. brucei* and other livestock trypanosomes.^13,14^ Results from this study suggest that vervet monkeys may lack trypanolytic factors, which have been reported to be present in other non-human primates, such as baboons and gorillas. The haptoglobin-related protein which has been demonstrated to have trypanolytic activity might be absent in the sera of vervet monkeys. In spite of the lack of human infection, *T. b. brucei* infections in rodents follow a similar pathogenesis to that of *T. b. rhodesiense*, thus rodents have been used as models for HAT.^8^ Some of the disadvantages of rodent HAT models include phylogenetic distance and an inability to perform sequential monitoring of CSF changes. These limitations can be addressed by using monkey models.

In the current study, infected vervet monkeys displayed classical clinical symptoms, parasitological and haematological trends that were similar to monkeys infected with *T. b. rhodesiense*.^15^ In addition, upon subcurative treatment with diminazene aceturate, disease relapse and re-emergence of blood parasitaemia took 85 days, which is similar to *T. b. rhodesiense* KETRI 2537 infection.^16^ However, the duration was longer than that reported for *T. b. rhodesiense* IPR 001 infections.^15^ The differences noted may be due to variation in parasite virulence.

Anaemia gives a reliable indication of the disease status and productive performance of trypanosome-infected animals.^17^ Disease in vervet monkeys tended to manifest with microcytic hypochromic anaemia during the early stages. This type of anaemia has been associated with iron deficiency that, in HAT, might arise from lack of incorporation into red cell precursors – despite the presence of adequate iron storage – and inefficient recovery of iron from the phagocytosed erythrocytes.^18,19^ Microcytic hypochromic anaemia has been described in vervet monkeys infected with *T. b. rhodesiense*.^20^ In this study, the severity of anaemia was greater in the acute stage of the disease (day 4 to 28 pi) than during the late stage (day 28 to 119 pi) and appeared to be associated with the level of parasitaemia. After curative treatment (119 dpi), all of the erythrocyte values recovered by 140 dpi because melarsoprol can clear parasites in all bodily compartments.^21^

There was a rapid decline in platelet counts in early-stage disease. Low platelet counts could be indicative of toxic products, originating from the trypanosomes, which cause platelet destruction.^18^ Splenic pooling of platelets and phagocytic removal of platelets by mononuclear cells has also been associated with thrombocytopaenia.^19^ Severe progressive thrombocytopaenia has been reported in *T. b. rhodesiense* vervet monkey models and human cases of sleeping sickness.^23,24^

Similarly to *T. b. rhodesiense* infections in vervet monkeys, lymphocytopoenia was noted in the early stage of the disease and lymphocytosis in the late stage.^17^

## Limitations of the study

Only three animals were used in this study; however an extensive amount of data was obtained. Parasitaemia was limited to the wet smear microscopic observation method only and, at very low parasitaemia, the method could have missed out early relapses. The characterisation of the model was also limited to haematological profiles and clinical examination.

### Recommendations

More extensive studies are needed to establish baseline levels of various biochemical parameters in vervet monkeys to aid in the determination of significant variations during infection/disease. Haematological profiles need to be studied in shorter sampling intervals (e.g. daily sampling for the first week of infection) in order to discern properly any changes during the acute disease phase.

## Conclusion

The clinical, parasitological and haematological observations obtained in this study compare well with those arising from *T. b. rhodesiense*; therefore, the vervet monkey *T. b. brucei* model may be used as substitute. This animal model may enable researchers and laboratory technicians to study HAT without the high risk of accidental infection.
